# Vehicle Deceleration Prediction Model to Reflect Individual Driver Characteristics by Online Parameter Learning for Autonomous Regenerative Braking of Electric Vehicles

**DOI:** 10.3390/s19194171

**Published:** 2019-09-26

**Authors:** Kyunghan Min, Gyubin Sim, Seongju Ahn, Myoungho Sunwoo, Kichun Jo

**Affiliations:** 1 Department of Automotive Engineering, Hanyang University, Seoul 04763, Korea; kyunghah.min@gmail.com (K.M.); bingju159@gmail.com (S.A.); msunwoo@hanyang.ac.kr (M.S.); 2 Department of Electronics and Controls, Hanyang University, Seoul 04763, Korea; gbcompany27@gmail.com; 3 Department of Smart Vehicle Engineering, Konkuk University, Seoul 05030, Korea

**Keywords:** vehicle speed prediction, driver behavior modeling, electric vehicle control, driver characteristics online learning

## Abstract

The connected powertrain control, which uses intelligent transportation system information, has been widely researched to improve driver convenience and energy efficiency. The vehicle state prediction on decelerating driving conditions can be applied to automatic regenerative braking in electric vehicles. However, drivers can feel a sense of heterogeneity when regenerative control is performed based on prediction results from a general prediction model. As a result, a deceleration prediction model which represents individual driving characteristics is required to ensure a more comfortable experience with an automatic regenerative braking control. Thus, in this paper, we proposed a deceleration prediction model based on the parametric mathematical equation and explicit model parameters. The model is designed specifically for deceleration prediction by using the parametric equation that describes deceleration characteristics. Furthermore, the explicit model parameters are updated according to individual driver characteristics using the driver’s braking data during real driving situations. The proposed algorithm was integrated and validated on a real-time embedded system, and then, it was applied to the model-based regenerative control algorithm as a case study.

## 1. Introduction

The prediction of vehicle states based on Intelligent Transportation System (ITS) information can be widely used to improve energy efficiency and driver convenience [1,2,3,4]. For electric vehicles, a smart regenerative control algorithm is an advanced driver assistance system which uses the prediction information of a vehicle decelerating from ITS information. This system recognizes the driving conditions that vehicle should decelerate by radar sensor. Then, it controls the regenerative torque of the electric motor automatically. This system provides the one-pedal driving technology, since the driver does not need to step on the brake pedal while accelerating using the acceleration pedal [5,6,7,8]. Thus, this autonomous braking system can serve driver convenience by reducing the driver’s pedal shifting. Furthermore, braking by using regeneration torque without the hydraulic braking can improve efficient energy management. However, the driver feels the heterogeneity due to this autonomous braking because each driver has different driving characteristics according to diverse driving situations. Thus, an appropriate prediction algorithm for vehicle states is required to generate the deceleration trajectory as the set point of this autonomous braking control on diverse deceleration conditions. Also, this algorithm should consider individual driver characteristics to apply this automatic braking system more practically [4,9,10,11,12,13,14].

In order to predict vehicle states, a number of studies on two methods have been typically introduced. The first method is a parametric model-based prediction. The intelligent driver model is a representative parametric model for vehicle-state prediction according to driver characteristics [14,15,16,17,18,19]. This model consists of a mathematical equation based on the physical behavior for car-following situations with some explicit parameters. The explicit model parameters are configurable variables, and these represent driver characteristics.

The other modeling approach is the data-driven method using the driving data of each driver. The deep-learning [20,21,22] and hidden Markov models [23,24,25] are representative prediction algorithms of data-driven methods. These algorithms can efficiently cover the modeling uncertainties and can provide accurate predictions. Due to the deep-learning algorithm’s advantages, much research has been conducted based on this algorithm.

The introduced research can predict the vehicle states with driver characteristics well. However, to apply the prediction algorithm for regenerative braking control, the algorithm should recognize diverse deceleration conditions such as stopping for a traffic light, slowing down for a speed bump, adhering to speed limits, or braking for a preceding vehicle. The algorithm should be specific for each deceleration condition. Furthermore, to reduce the sense of heterogeneity, the algorithm has to update the model parameters which represent the individual driver characteristics by online learning during real driving conditions. Most introduced data-driven algorithms require a premeasured data set to obtain the model and model parameters. Thus, typical data-driven methods are not appropriate for the online update algorithm.

In order to solve these problems, we proposed a parametric deceleration model and online learning algorithm for its parameters in an earlier work. The proposed model predicts the vehicle deceleration profile when the vehicle is braking in front of traffic lights. This model consists of mathematical equations; those equations are converted depending on the physical situation and explicit model parameters that represent individual driver characteristics. The online learning algorithm updates explicit model parameters during real driving conditions because the algorithm was designed to require less calculation time and memory for the real-time embedded system. However, the previous model was designed for only the deceleration condition for traffic light stops and the algorithm runs the prediction algorithm when determining deceleration start conditions.

**Figure 1:**
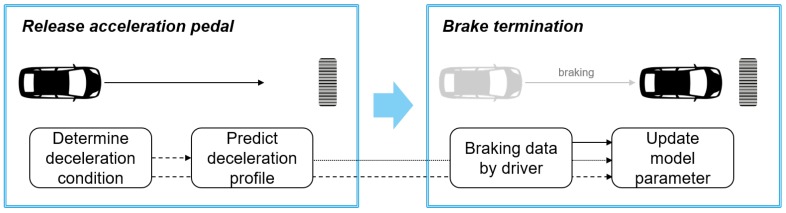
Algorithm operation process.

This paper proposes the extended parametric deceleration model and Deceleration Condition Recognition Algorithm (DCRA) to cover diverse deceleration conditions. The operation process of the proposed algorithm is described at Figure 1. When the driver releases the acceleration pedal, the DCRA determines whether the deceleration will occur in the future and the cause of the deceleration if deceleration will occur. According to the cause of the deceleration, the parametric deceleration model predicts the deceleration profile similarly to our previous research while the driver pushes the brake pedal. The online learning algorithm updates the model parameters using the prediction error between the predicted deceleration profile and the measured deceleration profile by the real driver when the braking ends. The updated model parameters are also managed according to the cause of the deceleration from the DCRA since the deceleration characteristics can vary depending on the cause of the deceleration as well as the driver.

To sum contributions of the paper up, the algorithm can predict the deceleration-specified profile based on the proposed parametric model structure and can represent the individual driver characteristics to the predicted deceleration profile for diverse deceleration conditions. The proposed algorithm was validated through processes in the loop simulation using real-driving data. Subsequently, we analyzed and classified individual driver characteristics using the explicit model parameters of validation results. Furthermore, based on the proposed algorithm, the regenerative control was conducted as a case study in the car-following deceleration conditions.

The rest of this paper is arranged as follows. Section 2 briefly introduces the system overview and the well-known intelligent driver model, which is applied as a frame structure of the proposed model. Section 3 presents the Deceleration Condition Recognition Algorithm (DCRA). According to the recognized deceleration condition, the prediction process of the model will be described in Section 4, and the online learning will be described in Section 5. Section 6 shows validation results through a vehicle experiment with regenerative control. The final section, Section 7, of this paper provides a conclusion.

## 2. System Architecture

### 2.1. Vehicle Configuration

**Figure 2:**
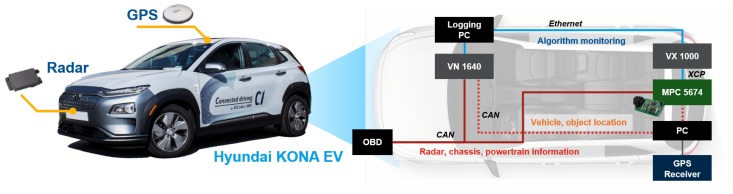
Vehicle configuration.

In order to acquire driving data for designing the proposed algorithm, a Hyundai KONA Electric Vehicle (EV) was used. The experimental vehicle was equipped with additional sensors and equipment to acquire ITS information, as the proposed algorithm requires precise information of the driving environment. To recognize the deceleration conditions at the car-following situation, the vehicle measures the preceding vehicle velocity and relative distance between the two vehicles using the equipped radar sensor, as described in Table 1. In addition, the algorithm should calculate precise positions of the ego-vehicle and other objectives to determine the deceleration condition by the road objectives in the driving environment. The low-cost Global Positioning System (GPS), high-definition (HD) map, and traffic-light recognition system are additionally installed in the vehicle to calculate the ego-vehicle position and to recognize road objective positions. Figure 2 shows the experimental apparatus of the vehicle. The proposed prediction model and learning algorithm are integrated on the embedded system, which is 32-bit microprocessor MPC5674 manufactured by NXP. Using the Vector VX1000, we have measured and calibrated the proposed algorithm through the universal measurement and calibration protocol (XCP). The additional personal computer calculates the relative distance between the vehicle and other objects. The calculated distance is transported to the embedded system through the Controller Area Network (CAN). Radar information and vehicle states which are measured by the vehicle-equipped sensor are also acquired through the CAN using a Vector VN1640 tool.

### 2.2. ITS Information Fusion

**Figure 3:**
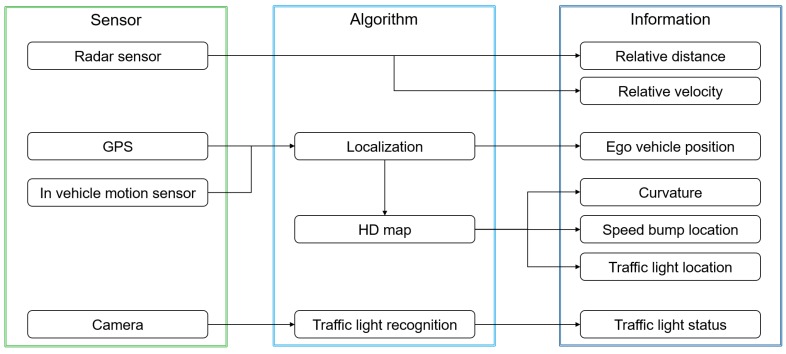
Intelligent Transportation System (ITS) fusion algorithm.

As mentioned in previous section, the precise positioning of the ego-vehicle and the static objectives are important to determine the deceleration conditions and, hence, to apply the prediction algorithm. To calculate the precise vehicle position, a localization algorithm which is introduced in References [26,27] is applied. The applied localization algorithm calculates the ego-vehicle position based on the GPS measurement position and in-vehicle motion sensor. On the other hand, the HD map provides the precise position of static objective and road curvature. Thus, the relative distance between the ego-vehicle and the static objective is calculated. Finally, the traffic-light recognition system classifies the traffic-light status based on the deep-learning algorithm that is introduced in Reference [28]. Figure 3 shows the algorithm architecture for ITS fusion.

### 2.3. Overall Structure of the Proposed Algorithm

**Figure 4:**
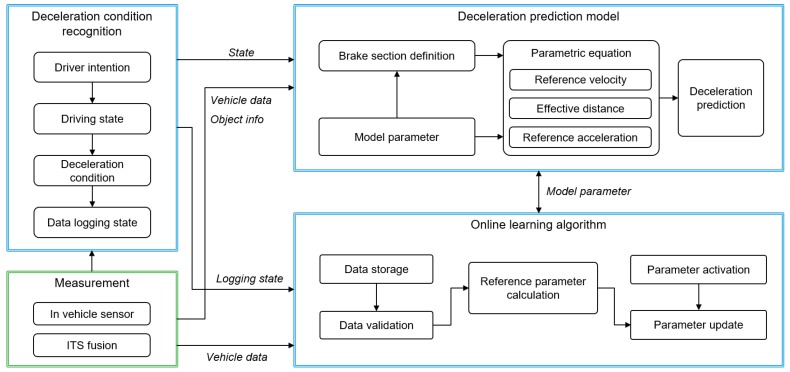
Overall algorithm structure.

The proposed algorithm consists of three parts, which can be seen in Figure 4. The Deceleration Condition Recognition Algorithm (DCRA) decides the driver intention and deceleration conditions depending on the cause of decelerating, which is measured by ITS information. According to the determined deceleration condition, a deceleration prediction model predicts the deceleration profile using the parametric equation with regard to the brake section and model parameters. After the prediction process ends, an online learning algorithm updates the model parameters using the measured deceleration data by the driver.

## 3. Deceleration Condition Recognition Algorithm

**Figure 5:**
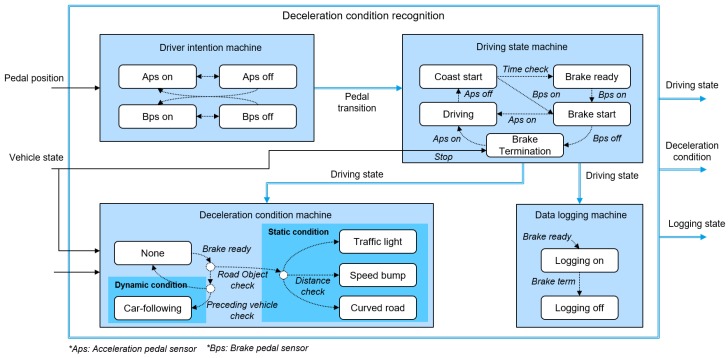
Diagram of the deceleration condition recognition algorithm.

In the urban driving condition, a vehicle faces diverse driving states [24,29]. To apply the proposed deceleration prediction model and learning algorithm appropriately, the future driving state is clearly defined. If the future driving state defines that the vehicle will decelerate, the cause of the deceleration is also identified to select the corresponding model parameters. Thus, the DCRA determines the driving state using the driver’s intention and the deceleration condition according to the cause of deceleration. The proposed condition recognition algorithm consists of four state machines as shown in Figure 5.

At first, the driver intention machine checks the driver’s pedal operation. When the driver presses or releases the accelerator pedal, the driver intention machine determines the pedal transition state as an accelerator pedal transition state. It also gives the brake pedal transition state for the brake pedal operation, and both the accelerator and brake pedal positions are measured by the in-vehicle network.

According to the pedal transition state of the driver intention machine, the driving state machine determines the driving state to affirm that the braking situation will occur. When the accelerator pedal is in the “off” state, the driving state machine sets the “coast start” state and checks the pedals’ off time. If the driver does not step on the accelerator pedal again during a specific period of time, the driving state machine will judge that the driving state is “brake ready”. This checking for pedal off-time can prevent the frequent state transition due to frequent acceleration. When the brake pedal is in the “on” state, the driving state machine gives a “brake start” state. The “brake termination” state then turns on when the driver releases their foot from the brake pedal. Finally, the state is determined as the “driving” state when the driver steps on the accelerator pedal.

The deceleration condition machine classifies the deceleration condition if the driving state is determined as a brake-ready state. The deceleration condition is classified according to the deceleration source, which causes the braking situation in a driving environment. In this study, we determine the deceleration conditions to have two categories: dynamic deceleration and static deceleration. The dynamic deceleration condition is a condition where a vehicle follows the preceding vehicle. This state is determined using dynamic information such as the relative velocity and the relative distance. Dynamic information comes from vehicle-equipped sensors such as the acceleration sensor, the wheel speed sensor, and the radar sensor. If the preceding vehicle decelerates so that the relative velocity is negative, the current deceleration condition is in a car-following deceleration state.

The static deceleration condition means the condition where the driver decelerates due to road objects on the driving route. The proposed algorithm selects three road objects: traffic lights, speed bumps, and road curvature. The algorithm can know the location of the traffic lights and speed bumps using ITS information like an HD map and GPS. Using this information, the distance to the road objects on the driving route can be calculated. Thus, when the driving state is brake ready and the relative distance to road objects are closer than configurable road-object distance criterion, the deceleration condition decides the vehicle will brake by the traffic light or the speed bump, respectively. The road curvature state is also another road object where a driver decelerates to reduce velocity to negotiate the curve without heterogeneity [30,31]. Similar to other road objects, the decision of the curve state is made using ITS information.

Finally, the data logging state is determined as the data logging judgment machine for the learning algorithm. When braking starts, this state machine gives that the data logging state is on. During the data logging on state, the vehicle data and ITS information are stored. Then, the learning algorithm updates the model parameter using the stored data after braking is terminated.

## 4. Deceleration Prediction Model

### 4.1. Prediction Model Description

The deceleration prediction will be conducted based on the well-known Intelligent Driver Model (IDM) [19]. This intelligent driver model can describe the microscopic car-following behavior using Equation (1). The vehicle acceleration is calculated according to several parameters such as the maximum acceleration $a_{m}$, the reference velocity $v_{ref}$, and the effective distance $d_{eff}$. In addition, the current vehicle velocity and relative distance of the preceding vehicle are also used in the model.

(1) $$a=a_{m}\left(1-{\left(v/v_{ref}\right)}^{\delta }-{\left(d_{eff}/d_{rel}\right)}^{2}\right)$$

**Figure 6:**
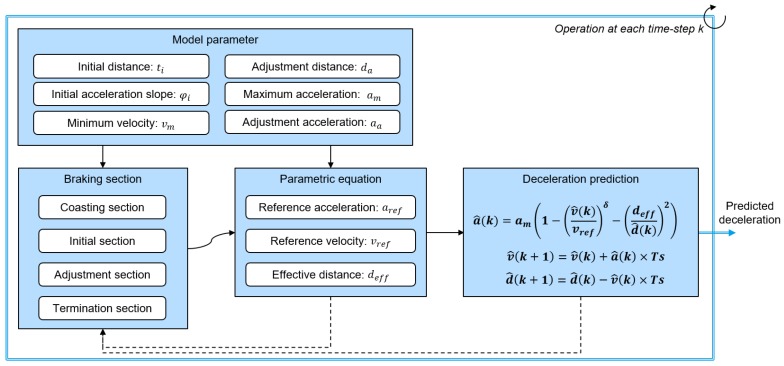
Diagram of the acceleration profile prediction algorithm.

In this paper, we proposed parametric equations for the reference velocity and effective distance to predict deceleration situations more specifically. Figure 6 shows the prediction process of the deceleration profile using the determined parametric equations. As shown in the figure, the parametric equations are determined depending on the braking sections and model parameters. Then, using this parametric equation, the deceleration profile $\hat{a}\left(k\right)$ is calculated at each time-step iteratively. The braking section is a time-range parameter which is determined using common patterns in a braking situation. The model parameters represent the individual driver’s characteristics explicitly and determine the parametric equation. Since parametric equations are defined differently according to each section and model parameters, the predicted deceleration profile that is a calculation result based on the intelligent driver model can minutely represent the deceleration characteristics depending on each section and driver characteristics. The detailed braking section and model parameters will be explained in the next section.

Furthermore, we proposed one more parametric equation that guarantees the end states for each of the deceleration conditions. That is a reference acceleration profile of Equation (2) which is calculated based on the constant acceleration model for location and velocity of the end of braking.

(2) $$a_{ref}\left(k\right)=0.5\left(v_{min}^{2}-v{\left(k\right)}^{2}\right)/d_{rel}\left(k\right)$$

where $a_{ref}$ is a reference acceleration, $v_{min}$ is the minimum velocity for each deceleration state, *v* is ego-vehicle velocity, and $d_{rel}$ is the relative distance to object. The minimum velocity is determined according to the deceleration conditions. In regard to the static deceleration condition, the minimum velocity and termination condition are predefined as a model parameter. In the traffic-light stop condition, the minimum velocity is determined as the zero value, while in the curve and speed bump conditions, the minimum velocity parameters are defined according to the road conditions. In the dynamic deceleration condition, the minimum velocity is the velocity of the preceding vehicle.

### 4.2. Braking Section Analysis

**Figure 7:**
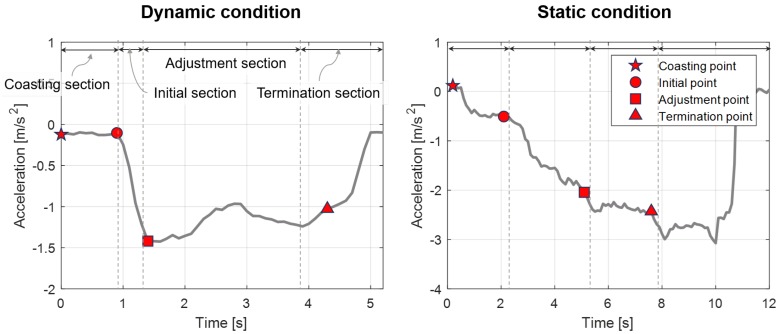
Description of the braking section about various deceleration conditions.

The deceleration data is analyzed to predict the deceleration profile based on drivers’ characteristics in each deceleration condition. Although the deceleration profile differs according to the driver’s driving style and deceleration conditions, the common feature in the declaration pattern is discovered. Therefore, we split the deceleration profile into four braking sections and its start points as shown in Figure 7. Details of each braking section are described as follows:

1. Coasting section: The coasting section is the time range from when a driver releases the accelerator pedal to when the driver pushes the braking pedal.
2. Initial section: In the initial section, the driver applies the brake pedal to reach the deceleration of the adjustment point. The driver decelerates with the jerk in this section. If the initial section is short and the jerk is large, the driver feels the rapid change of deceleration.
3. Adjustment section: In the adjustment section, the driver applies a specific braking pedal force to maintain the deceleration without abrupt changes. The pattern of deceleration in this section depends on the driver’s characteristic and the deceleration state.
4. Termination section: The termination section is the last part of the braking sections. Its definition also depends on driving conditions. In the static condition, the driver controls the braking pedal to satisfy the stop condition or minimum velocity in front of the static objectives. In the dynamic condition, the driver begins to remove the driver’s foot from the brake pedal.

### 4.3. Model Parameter Description

In order to represent the deceleration characteristics of the individual driver according to each determined braking section, explicit model parameters are determined. As mentioned in the previous section, these model parameters determine the time range of each braking section. The parameters also determine the parametric equations which are reference velocity and effective distance. Consequently, the vehicle acceleration in deceleration condition is predicted depending on the driver’s characteristics by model parameters affecting the braking section and parametric equations.

**Figure 8:**
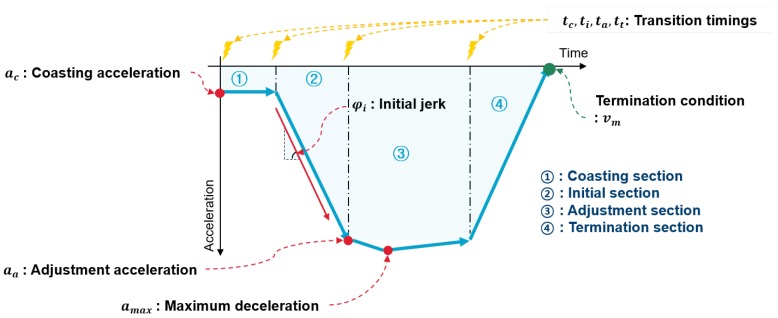
Model parameter description.

Figure 8 shows the model parameters in the deceleration. Transition-timing parameters are the transition timings of braking sections. According to the section transition, the parametric equations are also adapted. With regards to the acceleration value, the coasting acceleration and the adjustment acceleration parameters are the acceleration value when each section starts, and the minimum acceleration parameter is the negative maximum value during braking. This parameter is used as a maximum acceleration parameter at the classic IDM equation in Equation (1).

The initial acceleration slope parameter represents the jerk state when braking starts. Since the jerk can cause uncomfortable sensations, this parameter should be treated as an individual driver parameter. The minimum velocity determines the braking termination condition. About the static deceleration condition, the minimum velocity is defined as a model parameter according to each object. The minimum velocity for traffic-light conditions is zero because the vehicle should stop in front of the traffic light. The minimum velocity for curve and speed-bump conditions are determined depending on the driving conditions and the driver’s driving characteristics. On the other hand, the minimum velocity of the car-following condition is defined depending on the preceding vehicle velocity.

In addition, gain parameters determine the convergence of the predicted acceleration profile. When the braking progress is applied to the adjustment section, the acceleration profile converges to the reference acceleration because the driver follows the stop condition. The relative distance parameters affect the transition time from the initial section to the adjustment section. The detailed process of the braking section transition and transformation of parametric equations is described in the next section.

### 4.4. Calculation Process of the Parametric Model

**Figure 9:**
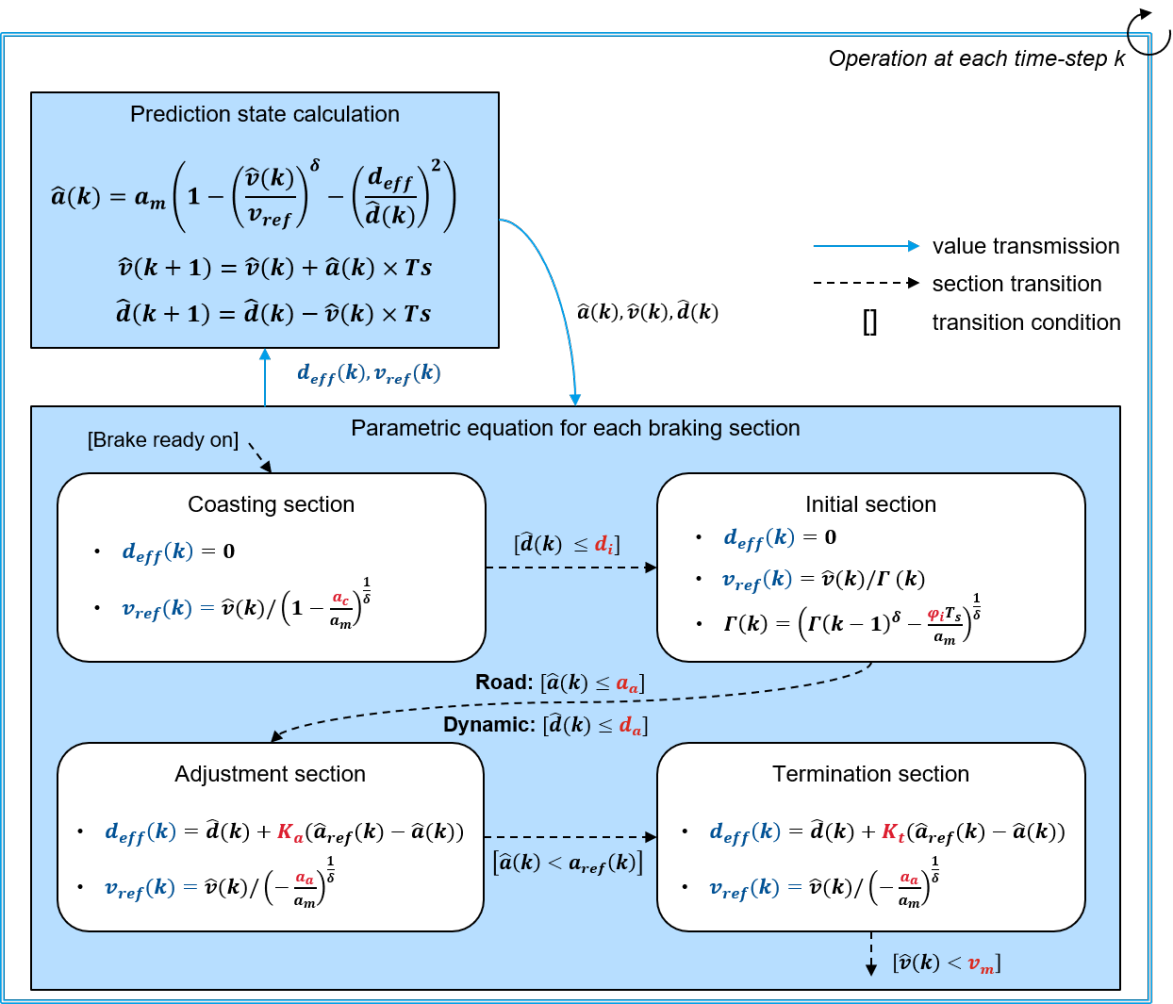
Detailed process for calculation of parametric model equations to predict deceleration profile.

As mentioned above, the deceleration prediction model calculates the deceleration profile using parametric equations of the reference velocity $v_{ref}$ and effective distance $d_{eff}$. These equations are determined using the model parameters according to the braking section as shown in Figure 9. As shown in the figure, parametric equations are determined differently to represent the specified deceleration characteristic of each braking section. At this time, the model parameters are applied to determine these equations. If the transition condition is satisfied, the prediction model shifts the braking section and the parametric equations are also changed. Model parameters also have an effect on the braking section transition as well as on determination of the parametric equations. The deceleration profile is calculated based on the modified intelligent driver model, and other predicted vehicle states about velocity and distance are calculated using this deceleration profile. Then, those predicted vehicle states are used to determine the parametric equations of the next step. This prediction process is operated iteratively while the prediction ends.

The descriptions of parametric equations for each braking section are explained as follows. In the coasting section, the model predicts the coasting deceleration with value $a_{c}$. The measured data shows that the deceleration values are around zero for all deceleration states. To continue this value, the value of the effective distance is determined as zero. Then, the reference velocity is updated according to the deceleration value as coasting starts.

In the initial section, the model predicts the jerk characteristics when braking starts. In this section, the effective distance is also set to zero, likewise in the coasting section. The reference velocity is calculated according to the acceleration slope φ of the model parameters. The parameters for each deceleration state determine the end time of the initial section. On the static deceleration condition, the drivers have a deceleration tendency to maintain the initial jerk until the deceleration reaches a specific deceleration value. Thus, the initial section is finished when the acceleration value reaches a specific acceleration value, which is an adjustment acceleration $a_{a}$. On the dynamic deceleration condition, the initial jerk is determined depending on the preceding vehicle state. Thus, the initial section of dynamic deceleration is finished when the relative distance is smaller than the adjustment relative distance $d_{a}$.

When the adjustment section starts, the driver normally adjusts the brake pedal to converge to the reference acceleration profile. To represent this convergence, the effective distance is determined using the acceleration error value between the predicted acceleration and the calculated reference acceleration profile. Since the acceleration is calculated by the ratio between the effective distance and relative distance, the initial value of effective distance is set to the adjustment relative distance. Then, the effective distance is adapted depending on the adjustment gain. This adjustment gain is defined differently depending on the respective deceleration conditions. The reference velocity is also calculated to keep the velocity ratio to the estimated velocity when the adjustment section starts. Finally, the adjustment section is finished when the acceleration reaches the reference acceleration.

When the braking is almost over, the driver controls the brake pedal to satisfy specific safety conditions. In the static deceleration conditions, the driver controls the acceleration to trace the reference acceleration to satisfy the minimum velocity at the front of the static object. On the other hand, the driver tends to keep a relatively safe distance according to the current vehicle speed of the car-following deceleration state. To guarantee these specific safety conditions, the model updates the effective distance similarly to the adjustment section using the termination gain parameter. When the vehicle velocity is slower than the minimum velocity or preceding vehicle velocity, the termination section is finished.

## 5. Online Learning Algorithm

### 5.1. Overview of Online Learning Algorithm

**Figure 10:**
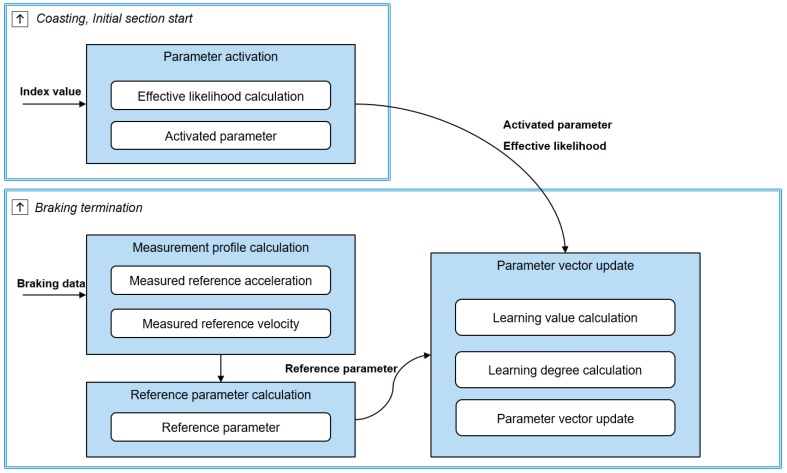
Process of the online learning algorithm.

Model parameters are used to determine parametric equations and braking-section transition of the deceleration-prediction algorithm. Thus, the deceleration-prediction algorithm predicts the various deceleration profiles depending on the parameter values. The proposed online learning updates these model parameters to reflect individual driver characteristics for various deceleration conditions. This algorithm consists of two parts; parameter activation and parameter update as shown in Figure 10.

The parameter-activation algorithm selects the parameter values of each model parameter using their learned vector arrays when deceleration starts according to the deceleration states. Then, the parameter-update algorithm updates the vector array value of each parameter using the reference parameter. The reference parameter is determined using the driver’s braking data after deceleration is finished.

### 5.2. Model Parameter Management

**Figure 11:**
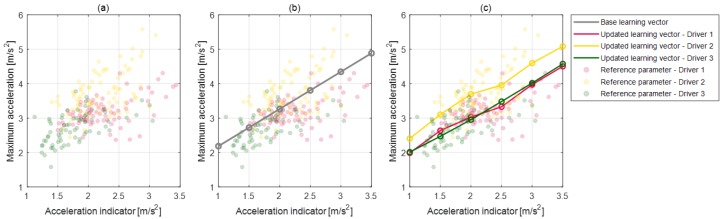
Maximum acceleration parameter and its updated vector array by the learning algorithm. (**a**: reference parameter value, **b**: based learning vector, **c**: updated learning vector.)

In order to manage parameters according to the deceleration states, the parameter values are defined as the vector array value depending on each vector index. The deceleration state which affects the selection of the parameter value is defined as the vector index of the vector array such as the initial deceleration condition, relative distance to the object, or ego-vehicle velocity. This vector index for each parameter is determined as a correlated deceleration state to its model parameter value. Thus, the parameter activation algorithm can select appropriate parameter values depending on the deceleration states. For example, a maximum acceleration parameter is highly correlated to the coast acceleration indicator for all drivers, which can be seen in Figure 11a. The coast acceleration indicator is determined as the difference between the reference acceleration value and the vehicle acceleration value when the coast section starts and is given in Equation (3). This parameter represents the deceleration start states due to the larger value of this parameter, which suggests that the driver should brake more strongly to satisfy the deceleration criterion. Therefore, the parameter activation algorithm should select the large maximum acceleration parameter value to predict strong deceleration if the deceleration state is a large coast acceleration indicator. By determining the vector array value and its index for the maximum acceleration parameter as shown in Figure 11b, the parameter activation algorithm can select the appropriate parameter value of the maximum acceleration according to the deceleration start state. Then, the online learning algorithm updates the parameter vector array value using the measured braking data of the individual driver when braking terminates. At this time, the updated value of the parameter vector is also related to the index indicating the deceleration states of braking data. As a result, the proposed algorithm learns and reflects on the individual driver’s driving style according to various deceleration states, which can be seen in Figure 11c.

(3) $$a_{ind,cst}=a\left(t_{c}\right)-a_{ref}\left(t_{c}\right)$$

**Figure 12:**
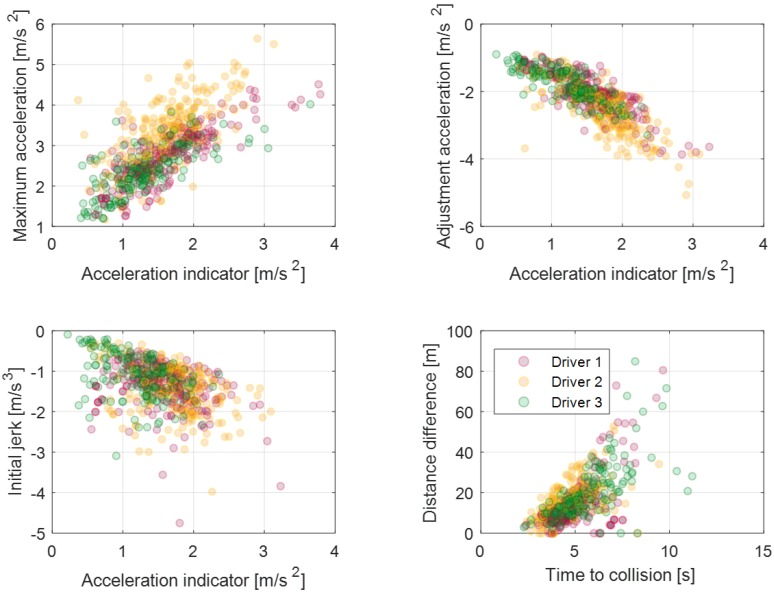
Model parameters and parameter indices for static deceleration condition.

Figure 12 shows the model parameters and their indices for static deceleration conditions. As mentioned in the explicit model parameters section, model parameters which are related to the value of acceleration represent the driver’s characteristics explicitly. Thus, we determined two model parameters about the acceleration value: the maximum acceleration parameter and the adjustment acceleration parameter. As mentioned above, the maximum acceleration parameter is correlated to the coast acceleration indicator, which represents the start condition of deceleration. Similarly, the adjustment acceleration parameter is correlated to the initial acceleration indicator, which represents the deceleration state when the driver starts to step on the brake pedal.

The initial jerk parameter, which is an acceleration differential value at the initial section, is also defined as a model parameter with the index of the initial acceleration indicator. As shown in Figure 12, the driver tends to decelerate strongly with larger adjustment acceleration and the initial jerk when the acceleration indicator is large like the maximum acceleration parameter. The last parameter is a distance difference from the coasting section to the initial section. This parameter is correlated with the time-to-collision value of coasting start. It represents that the driver’s pedal-shift time decreases as the value of time to collision decreases.

**Figure 13:**
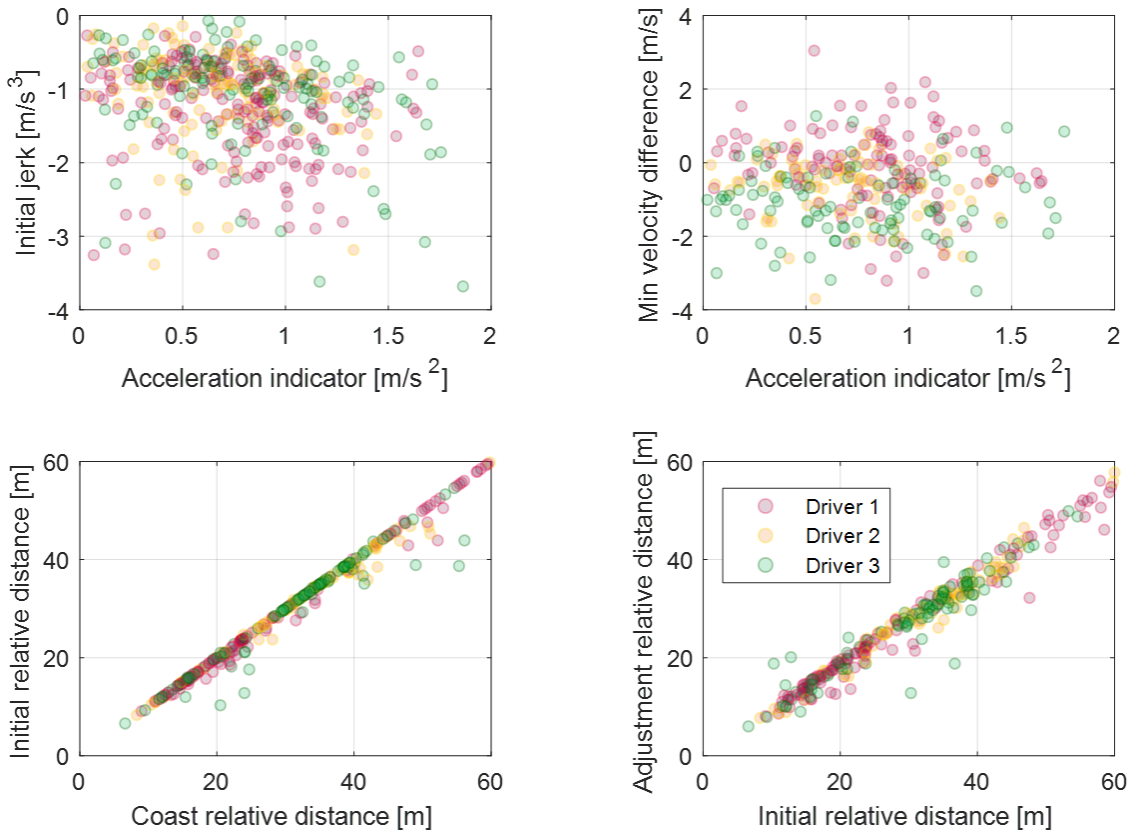
Model parameters and the parameter indices for dynamic deceleration condition.

In the dynamic deceleration condition, a driver’s driving style is affected based on the preceding vehicle behavior. Therefore, the model parameters are determined as relative states from the preceding vehicle. The relative distance of the initial section is determined by the relative distance of the coast section as shown in Figure 13. This denotes that the driver tends to keep a relative distance during a car-following situation. Subsequently, the vector index of each relative distance parameter is determined by the relative distance of the previous braking section. The initial jerk parameter is also important for the dynamic deceleration condition. However, the correlation with the acceleration index is lower than the correlation of the static dynamic condition because the acceleration index be affected by preceding vehicle behavior. The minimum velocity difference is a velocity difference between the ego-vehicle’s minimum velocity and the preceding vehicle’s minimum velocity during the deceleration. This parameter does not have a large correlation with the acceleration index, but the average value can represent drivers’ characteristics about the deceleration termination condition.

### 5.3. Parameter Activation

To use the model parameters for the prediction model, the parameter activation algorithm selects the parameter value of each parameter from the its parameter vector array value. The selected parameter value should be related according to the relevant deceleration states. In addition, the parameter value is treated as stochastic because the parameter vector is updated based on the data-driven method [32].

As mentioned above, the vector index is correlated with the parameter vector and represents deceleration states. Thus, the parameter activation algorithm selects the parameter value by selecting the highly correlated vector index to the current deceleration states. To obtain the degree of relation between the vector index and deceleration states, an effective likelihood is determined by normalizing the Gaussian distribution for each index value as can be seen in Equation (4).

(4) $$P\left(i\right)=Norm\left(\frac{1}{\sigma \sqrt{2\pi }}\exp^{-\frac{1}{2}{\frac{\left(i-i_{val}\right)}{\sigma }}^{2}}\right)$$

where P(i) is the effective likelihood for each vector index *i*, $i_{val}$ is the index value for the current driving state, and σ is the standard deviation of the index value.

**Figure 14:**
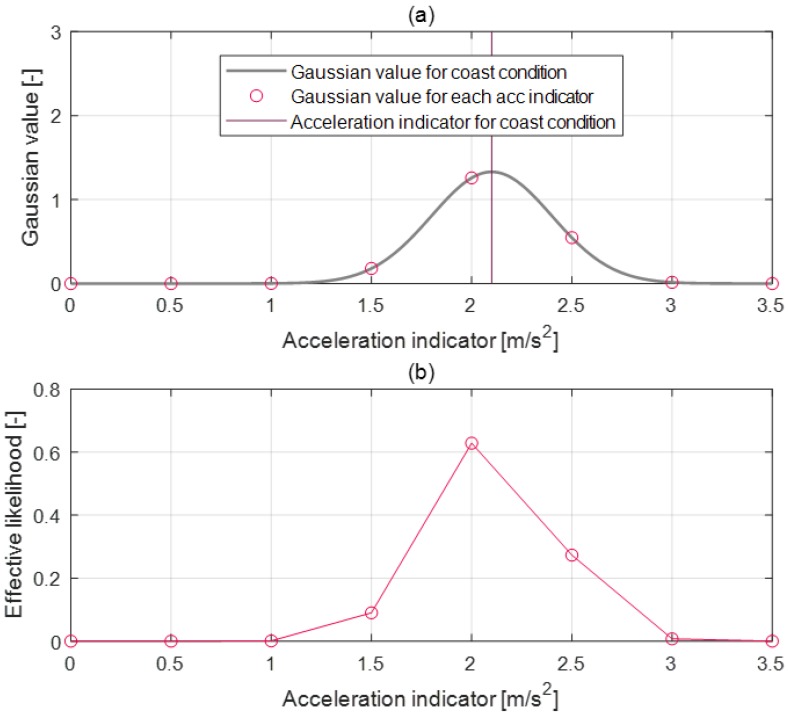
Gaussian value and effective likelihood according to acceleration indicator value. (**a**: Gaussian value, **b**: Effective likelihood).

For example, when the acceleration indicator is calculated as a 2.1 value according to the deceleration states as shown in Figure 14a, the effective likelihood of the vector index is also determined based on the Gaussian distribution as shown in Figure 14b. The effective likelihood of acceleration indicator value 2 and 2.5 are larger values than the others because the acceleration indicator of the current deceleration state is a 2.1 value. Then, the parameter value is determined by inner product of the effective likelihood and parameter vector value set as shown in Equation (5). Through this process, the parameter-activation algorithm can select the parameter value that is correlated with current deceleration states to use for the deceleration prediction model.

(5) $$\theta_{act}=P\left(i\right)•V_{\theta }\left(i\right)$$

where $\theta_{act}$ is an activated parameter and $V_{\theta }\left(i\right)$ is a vector array value for parameter θ.

### 5.4. Reference Parameter Calculation

In order to update the vector value of each parameter to reflect individual driver characteristics, the online learning algorithm calculates the reference values of each parameter using the braking data of the driver. At first, the transition points of each braking section are determined because the proposed model parameters are determined related to the braking section transition. For example, the adjustment acceleration is determined as the acceleration value when the adjustment section starts. The initial jerk is also determined using the acceleration values between the start point of the initial section and start point of the adjustment section.

**Figure 15:**
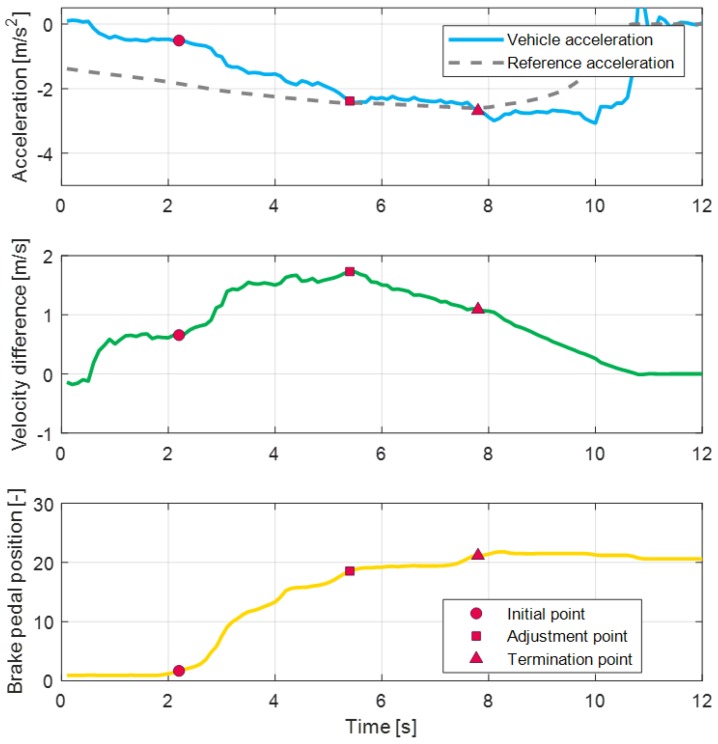
Detailed process for calculation of parametric model equations to predict deceleration profile.

The transition times for the coasting section and the initial section are determined easily using the pedal transition information of the driver. When the driver releases the acceleration pedal, the coasting section starts. Then, the initial section starts when the driver pushes the brake pedal. To determine the transition times for the adjustment section and termination section, the reference acceleration and reference velocity are calculated using the measured braking data. These two profiles are described in the prediction model section. The reference velocity is calculated based on the IDM equation to find the adjustment point. By setting the effective distance as a zero value and by using the minimum velocity parameter, the reference velocity profile $v_{ref,measure}$ is defined as Equation (6). Then, the velocity difference is calculated between the reference velocity and the measured velocity, as shown in Figure 15. As shown in the figure, the adjustment point can be determined as the maximum velocity difference point. The timing point is determined as the termination point when the vehicle acceleration is converged to the measured reference acceleration.

(6) $$v_{ref,measure}\left(k\right)=\left(v\left(k\right)-v_{min}\right)/\left({1-\frac{a(k)}{a_{m}}}^{\frac{1}{\sigma }}\right)$$

Reference variables of relative distance parameters are defined by using the measured relative distance from the radar sensor at each section transition point. The reference value of the minimum velocity parameter is defined using the measured vehicle velocity during the braking conditions. Similarly, the reference value of maximum acceleration is also defined using the measured vehicle acceleration.

### 5.5. Parameter Vector Update

After calculation of the reference value using the braking data, the online learning algorithm updates the vector array value. The error value is calculated using the activated parameter value $\theta_{act}$ from the previous vector array value and the reference value $\theta_{ref}$ from the braking data. Then, the error value defines an update target value $\delta_{\theta }$ with a learning rate α as Equation (7). Through the simple value incremental learning process [33], the update algorithm adds the update values to each vector value as Equation (8).

(7) $$\delta_{\theta }=\alpha \left(\theta_{ref}-\theta_{act}\right)$$

(8) $$V_{\theta }{\left(i\right)}^{+}=V_{\theta }{\left(i\right)}^{-}+\psi \left(i\right)\delta_{\theta }$$

In this process, the update algorithm applies the learning degree ψ(i), which is a weight value to determine the updating weights for each vector value. Similar to the parameter activation, the parameter vector value, which is highly correlated to the current driving status, should be updated with a large update weight. Thus, the learning degree is determined based on the effective likelihood to consider the current deceleration states for model parameter as displayed in Equations (9) and (10). Equation (9) is derived from Equations (5) and (8). It means that the activated parameter value using the updated vector array value $P\left(i\right)•V_{\theta }{\left(i\right)}^{+}$ should be the same as the reference parameter value $\theta_{ref}$ if the deceleration states are the same. Using these equations, the learning degree resolves the value of parameter vector to be updated correctly. In addition, the learning degree leads the larger update value for the larger effective probability.

(9) $$\psi \left(i\right)={\left(\sum P\left(∼i\right)\times \sum \left(\frac{P(i)}{\sum P(∼i)}\right)\right)}^{-1}$$

(10) ∑P(∼1)=P(2)+P(3)+⋯+P(8)

## 6. Vehicle Experimental Results

### 6.1. Experimental Condition

The proposed algorithm was validated through the vehicle experiment. To validate the static deceleration condition, the algorithm has to know the exact position of the objectives and its status. Since the predefined HD map can provide the position of objectives, the validation experiment for the static deceleration condition was conducted at a specific test cite. The proving ground is Yeongjongdo in Incheon, South Korea, whereby this site contains a speed bump, traffic light, and curvature road conditions to successfully carry out experiments. The algorithm for the dynamic deceleration condition in the car-following situation was validated in various driving conditions for urban driving and highway-driving environments. To specify the driving characteristics for the individual driver, three drivers conducted the vehicle experiments according to the case listed above. The purpose of the proposed research is to present a predictive model for various deceleration conditions and to explicitly update the driving characteristics of individual drivers and not to macroscopically classify them using machine learning methods. Therefore, even if a model is defined and validated using a small number of driver’s data, the model is valid if it can clearly show the individual driver’s characteristics for various deceleration conditions.

### 6.2. Deceleration Prediction Results Under Various Driving Conditions

**Figure 16:**
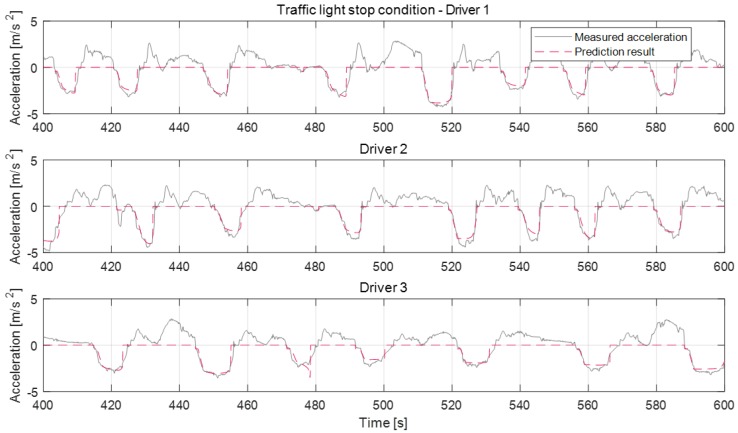
Deceleration prediction results on traffic-light stop condition.

**Figure 17:**
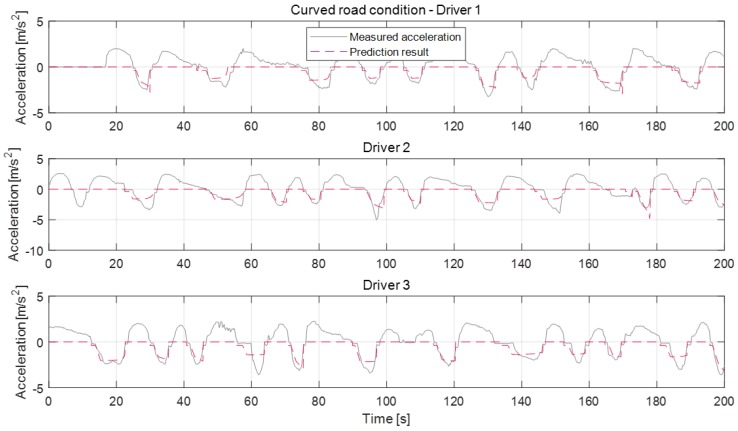
Deceleration prediction results on curved road condition.

**Figure 18:**
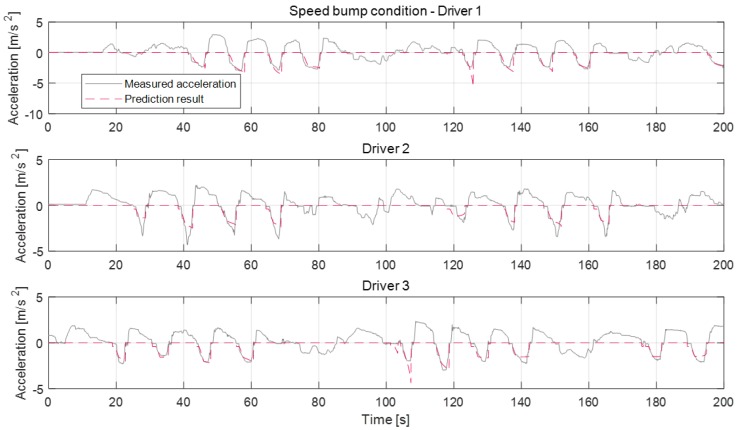
Deceleration prediction results on speed bump condition.

Figure 16, Figure 17 and Figure 18 show the deceleration prediction results of the model in static deceleration conditions. The static deceleration condition contains the traffic-light stop condition, curved road condition, and speed bump condition. About the static deceleration condition, the model parameters were respectively updated according to individual driver for each deceleration condition. Therefore, the proposed model can predict the deceleration profiles depending on the deceleration conditions, as shown in figures. The prediction results about the traffic-light stop condition are more accurate than the curved road condition and speed bump condition. Since the termination velocity parameter affects the prediction accuracy, the traffic-light stop condition which determines the termination velocity parameter as the fixed zero-velocity value shows a more accurate prediction than other conditions. About curved road conditions, the termination velocity is determined according to the maximum road curvature and its position from the ego-vehicle. However, since the termination velocity is not constant, even though the max curvature and driver are same, it is difficult to determine the exact termination velocity. Therefore, the prediction error that occurred in some deceleration cases of curve condition by the inaccurate activation of the termination velocity parameter, such as the deceleration case of about 50 s for driver 1 or of about 60 s for driver 3. The deceleration profiles about the speed bump condition show similarities to the stop case. According to the tests, driver 2 tends to decelerate more aggressively. Especially for the bump case, the maximum deceleration values of driver 2 are almost near 5 m/s^2^. The prediction model cannot represent this strong braking at an early braking state. The model does not work at deceleration cases around 100 s of bump condition because the deceleration occurs due to other reasons and not just due to the speed bump.

**Figure 19:**
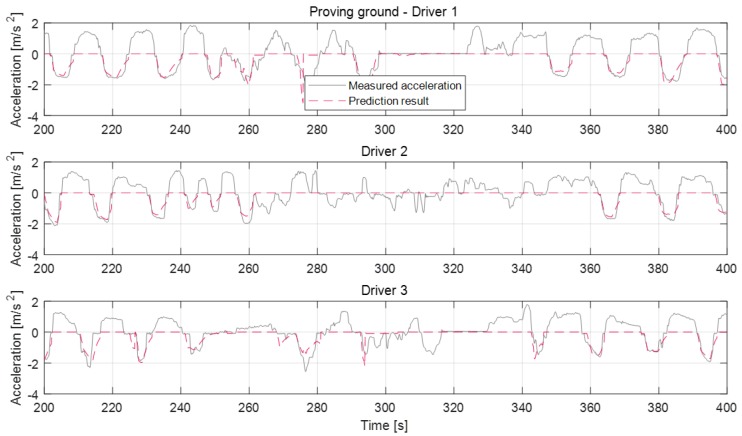
Deceleration prediction results in the dynamic deceleration condition on proving ground.

The deceleration model about dynamic conditions was validated through proving ground driving and real road driving. Real road driving contains various urban and highway driving environments. In the proving ground results, the vehicle deceleration occurred by deceleration of a preceding vehicle. The results shown in Figure 19 are consistent in the stable deceleration as the preceding vehicle decelerated intentionally. The prediction results show also that the model predicts the deceleration profile at each deceleration case though the preceding vehicle affects deceleration as well. The model did not predict the deceleration on some deceleration cases because the vehicle deceleration was not caused by the preceding vehicle. The prediction about deceleration cases for around 280 s for driver 1 or 290 s for driver 3 are terminated even before the deceleration ends. This prediction termination occurred because the preceding vehicle deviated from the driving course.

### 6.3. Parameter Learning Results

As mentioned in the parameter management section, the model parameter values are managed as vector values according to the highly correlated index. At first, using the vehicle experiment data for all drivers, we determined the base parameter vector to apply the proposed learning algorithm. As shown in the algorithm overview in Figure 4, the proposed algorithm updates the parameter vector when each deceleration is terminated. This process was concurrent with the verification of the prediction algorithm through the various experiment data that are described in the previous prediction validation section.

**Figure 20:**
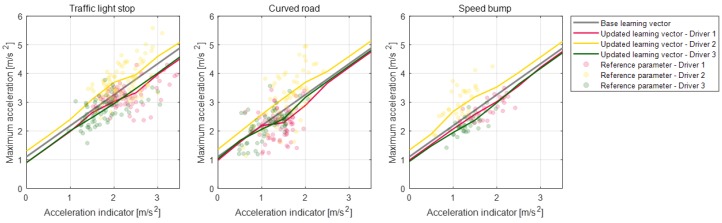
Learning results for parameter maximum acceleration for each road object.

**Figure 21:**
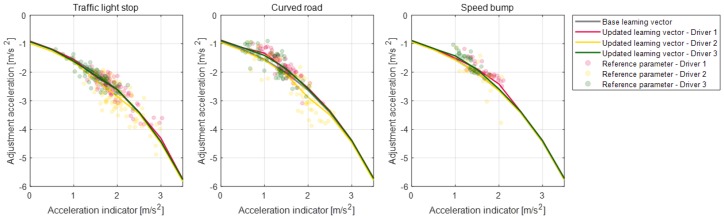
Learning results for parameter adjustment acceleration for each road object.

**Figure 22:**
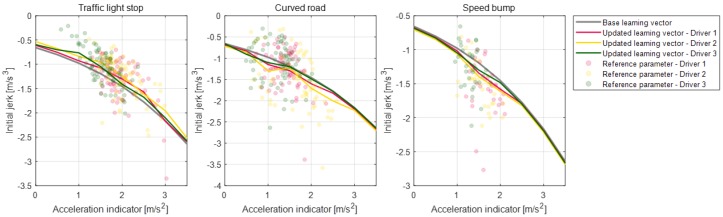
Learning results for parameter initial jerk for each road object.

**Figure 23:**
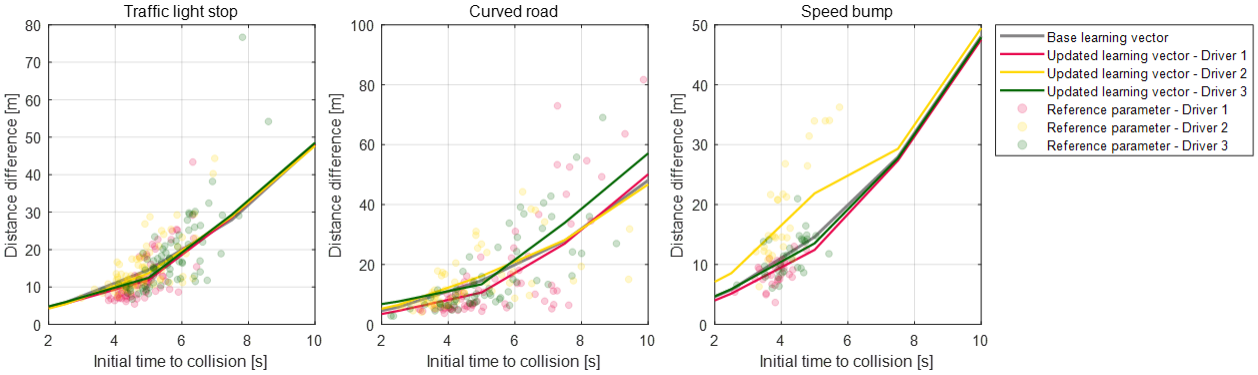
Learning results for parameter distance difference for each road object.

Parameter vectors of the static condition deceleration model are updated according to each deceleration condition for the individual drivers. Figure 20, Figure 21, Figure 22 and Figure 23 depict the learning results of the static model parameters: maximum acceleration, adjustment acceleration, initial jerk, and distance difference. Those updated results for the model parameters can represent the driver characteristics on each deceleration condition. As shown in Figure 20, the updated learning vectors about the maximum acceleration parameter shows that, generally, driver 2 decelerates with larger deceleration values than the other drivers in any deceleration conditions. Similar driver characteristics are also described as the adjustment acceleration parameter and initial jerk parameter in Figure 21 and Figure 22. The learning results of these parameters on driver 2 have larger negative values than the learning results of the other drivers. These results could mean that the driving characteristic of driver 2 is more aggressive than the other drivers. The initial jerk and the distance difference parameters represent the different characteristics according to not only the driver but also the deceleration condition. The initial jerk parameter which affects the deceleration feeling in early deceleration is larger for the traffic-light stop condition than for other deceleration conditions.

**Figure 24:**
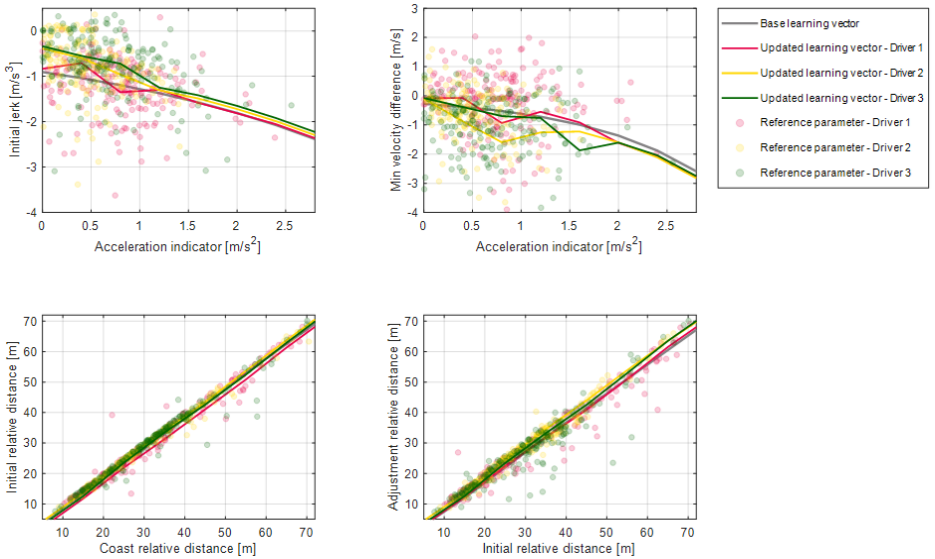
Learning results for model parameters on the dynamic deceleration condition.

Figure 24 shows the updated results of the parameter vectors about the dynamic deceleration condition. According to the drivers’ results, driver 1 shows deceleration characteristics with a larger initial jerk than the others. These results show that driver 1 pushes the brake pedal with more strength when braking starts. The updated learning vector on the minimum velocity difference parameter of driver 1 is larger than of the other drivers. It means that driver 1 is slower than other drivers when the driver terminates deceleration. The updated learning vector on the initial relative distance parameter of driver 1 is smaller than of the other drivers. This suggests that driver 1 uses more time to pedal shift from the accelerator pedal to the brake pedal. Learning results about the adjustment relative distance show a similar tendency with the initial relative distance parameter for each driver.

### 6.4. Case Study to Prediction Model-Based Regenerative Control

**Figure 25:**
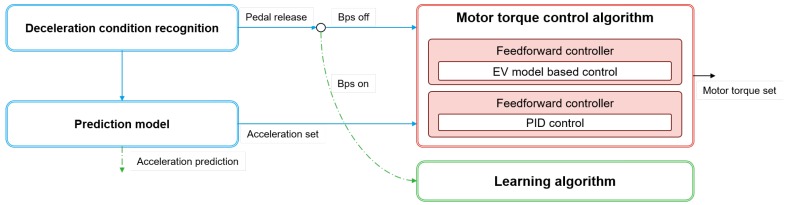
Structure of the regenerative controller based on the proposed driver model.

The proposed algorithm was applied to the smart regenerative control system of an electric vehicle as a case study. The controller structure is shown in Figure 25. The deceleration condition recognition algorithm determines the vehicle deceleration when the driver releases their foot from the acceleration pedal. Afterwards, the motor torque control algorithm generates the regenerative torque if the driver does not push the brake pedal. At this time the acceleration profile from the prediction model is used as the acceleration set point of the motor torque control algorithm. On the other hand, the motor torque control algorithm does not operate if the driver pushes the brake pedal. In this case, the prediction model just predicts the acceleration profile; then, the learning algorithm updates the parameter vectors using the braking data.

The torque control algorithm consists of two controllers: the feedforward controller and the feedback controller. The feedforward controller determines the motor torque based on the electric vehicle model. Using the electric vehicle model, the feedforward controller calculates the desired regenerative motor torque to generate the acceleration set point. The feedback controller is a well-known Proportional-Integral-Derivative controller (PID controller) to trace the acceleration set point from the prediction model.

**Figure 26:**
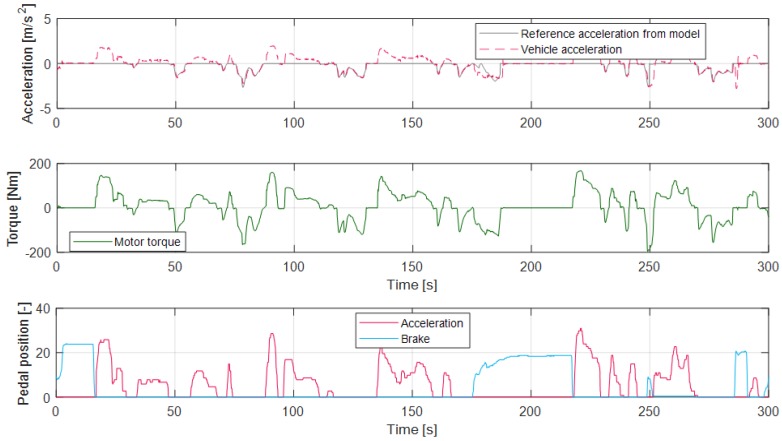
Deceleration prediction model-based regenerative control results.

The Vehicle Control Unit (VCU) controls the motor torque according to the generated regenerative torque from the control algorithm. Consequently, the vehicle decelerates without the driver’s braking pedaling action. Thus, it provides driving convenience by excluding pedaling of the vehicle’s brake. Figure 26 shows the deceleration control results using the proposed driver model and the torque controller; the red dashed line in the top graph is the actual vehicle acceleration, and the gray solid line is the predicted acceleration profile from the model. As shown in the figure, vehicle acceleration traces the acceleration set point from the model from 40 s to 170 s and around 230 s. In contrast, the model only predicts the acceleration profile when the driver pushes the brake pedal around 200 s. At this time, the vehicle is decelerated according to the driver’s braking action and the learning algorithm updates the model parameter vectors.

## 7. Conclusions

In this paper, we proposed a deceleration prediction model based on individual driver characteristics. The proposed prediction model is designed based on the mathematical intelligent driver model, and it is modified especially to the deceleration prediction with some explicit model parameters. First, we defined the braking section that describes the deceleration characteristics to specifically predict the deceleration state. Then, the parametric equations were calculated according to the braking section with explicit model parameters to predict the deceleration profile. The model parameters represent the individual driver characteristics by updating the vector array values online. These vectors are also updated according to the various deceleration conditions about the various road objects or preceding vehicle. Thus, the proposed model can be used for various deceleration conditions by considering the individual driver’s characteristics. The proposed algorithm was validated through vehicle experiments and applied to smart regenerative control. Since the model can execute various deceleration conditions and driver characteristics, the smart regenerative control based on the proposed model does not cause the sense of heterogeneity for an individual driver. In future research, the proposed algorithm will be applied to smart regenerative systems on real driving situations. To achieve this, the deceleration condition recognition algorithm will be modified more practically. The algorithm can substitute the navigation device for the HD map and GPS to determine information of road objectives. Furthermore, the dynamic deceleration condition also is extended to consider traffic jam or cut-in situations.

## Figures and Tables

**Table 1:** Sensor specification.

Sensor	Specification
Radar	Maximum range: 150 m
	FOV: +/− 10 degrees over 60 m, +/− 45 degrees under 60 m
	Update rate: 50 ms
GPS	Accuracy (Root mean square): 2.5 m
	Update rate: 20 ms
